# Blocking CCR5 activity by maraviroc augmentation in post-stroke depression: a proof-of-concept clinical trial

**DOI:** 10.1186/s12883-024-03683-3

**Published:** 2024-06-06

**Authors:** Oren Tene, Jeremy Molad, Ofer Rotschild, Aviva Alpernas, Muhamad Hawwari, Estelle Seyman, Nir Giladi, Hen Hallevi, Einor Ben Assayag

**Affiliations:** 1 Department of Psychiatry, Tel Aviv Sourasky Medical Center, Tel Aviv, Israel; 2 Department of Neurology, Tel Aviv Sourasky Medical Center, Tel Aviv, 64239 Israel; 3 Department of Psychiatry, Faculty of Medicine, Tel Aviv University, Tel Aviv, Israel; 4 Department of Neurology-Stroke, Rambam Medical Center, Haifa, Israel; 5 Brain Institute, Tel Aviv Sourasky Medical Center, Tel Aviv, Israel; 6 Department of Neurology, Faculty of Medicine, Tel Aviv University, Tel Aviv, Israel; 7 Sagol School of Neuroscience, Tel Aviv University, Tel Aviv, Israel

**Keywords:** Post-stroke depression (PSD), CCR5 antagonist, Maraviroc, Drug repurposing, Clinical trial

## Abstract

**Background:**

Post-stroke depression (PSD) is a significant impediment to successful rehabilitation and recovery after a stroke. Current therapeutic options are limited, leaving an unmet demand for specific and effective therapeutic options. Our objective was to investigate the safety of Maraviroc, a CCR5 antagonist, as a possible mechanism-based add-on therapeutic option for PSD in an open-label proof-of-concept clinical trial.

**Methods:**

We conducted a 10-week clinical trial in which ten patients with subcortical and cortical stroke, suffering from PSD. were administered a daily oral dose of 300 mg Maraviroc. Participants were then monitored for an additional eight weeks. The primary outcome measure was serious treatment-emergent adverse events (TEAEs) and TEAEs leading to discontinuation. The secondary outcome measure was a change in the Montgomery-Asberg Depression Rating Scale (MADRS).

**Results:**

Maraviroc was well tolerated, with no reports of serious adverse events or discontinuations due to intolerance. The MADRS scores substantially reduced from baseline to week 10 (mean change: -16.4 ± 9.3; *p* < 0.001). By the conclusion of the treatment phase, a favorable response was observed in five patients, with four achieving remission. The time to response was relatively short, approximately three weeks. After the cessation of treatment, MADRS scores increased at week 18 by 6.1 ± 9.6 points (*p* = 0.014).

**Conclusions:**

Our proof-of-concept study suggests that a daily dosage of 300 mg of Maraviroc may represent a well-tolerated and potentially effective pharmacological approach to treating PSD. Further comprehensive placebo-controlled studies are needed to assess the impact of Maraviroc augmentation on PSD.

**Trial registration:**

ClinicalTrials.gov Identifier: NCT05932550, Retrospectively registered: 28/06/2023.

**Supplementary Information:**

The online version contains supplementary material available at 10.1186/s12883-024-03683-3.

## Introduction

Depression is a frequent neuropsychiatric complication of brain ischemia, associated with increased mortality, higher functional disability, lower quality of life and a greater risk for cognitive decline [1–3]. Accumulating evidence suggests that inflammatory processes and neural-immune interactions are involved in post-stroke depression (PSD) [4]. Related theories focus on synaptic plasticity as an important means of recovery from both diseases after converged molecular and cellular mechanisms, including inflammation causing atrophy of neurons, loss of glutamatergic synaptic connections, and dysfunction of the circuitry that is essential for mood regulation and cognitive function [5]. Indeed, PSD can be perceived as a psychoneuroimmunological disorder in which inflammatory mechanisms involving cytokines and chemokines play a crucial role. Both acute stroke and depressed patients consistently show higher levels of pro-inflammatory cytokines, acute phase proteins, chemokines and cellular adhesion molecules [6, 7].

The C-C chemokine receptor type 5 (CCR5) is involved in immune processes and neuroplasticity and is highly expressed in T cells and macrophages, as well as microglia, astrocytes, and neurons, in multiple brain regions [8]. CCR5 is a pro-inflammatory receptor and inhibition of its signaling has been shown to enhance plasticity processes in hippocampal and cortical circuits. Previous studies show that inhibiting CCR5 activity reduced the activation of glial cells, preserved the integrity of endothelial cell layers, reduced the infiltration of T cells, and decreased neuroinflammation [9]. Moreover, in a recent study conducted by our group and others, CCR5 blockade significantly promoted axonal sprouting in the pre-motor cortex after experimental stroke [10]. In humans, we have evaluated the effects of the loss-of-function mutation known as CCR5-Δ32 (which impairs, at least partially, the function of the CCR5 receptor) in 435 post-stroke patients (the TABASCO prospective cohort study). In patients who were carriers of the CCR5-Δ32 allelle (about 15% of the cohort), we found less depressive, anxiety, and post-traumatic symptoms 6, 12, and 24 months after their stroke, aside from showing a significantly better cognitive and functional outcome. These findings implied that impaired activity of the CCR5 may have therapeutic advantages in PSD recovery, and pointed to a mechanism-based treatment target for post-stroke depression, as drugs mimicking the action of this loss of function mutation are available [11].

On this basis, we performed a proof of concept open-labeled clinical trial using Maraviroc (Selzentry), the Food and Drug Administration (FDA) approved CCR5 antagonist for R5-tropic HIV-1 infected patients, as a novel add-on intervention for PSD.

Maraviroc is a small molecule administered orally, metabolized by CYP3A4 [12]. It has a good pharmacokinetic profile, relatively low protein binding, and high bioavailability [13, 14]. The drug has been available in the US market since 2007 and has a known and satisfactory safety profile. Main adverse events include upper respiratory tract infections, cough, pyrexia, and rash [15].

We hypothesized that Maraviroc would be a safe and effective add-on treatment option for patients suffering from PSD.

### Primary objective

To investigate the safety and tolerability of Maraviroc 300 mg per day in subjects with PSD.

### Secondary objective

To evaluate the effect of Maraviroc 300 mg per day on improving depressive symptoms in subjects with PSD, as assessed by a change from baseline in the Montgomery-Asberg Depression Rating Scale (MADRS) total score and in improving cognitive, functional, and behavioral outcomes.

## **Methods**

### Protocol approvals and patient consent

The study protocol (see Supplementary material) was approved by the Tel Aviv Sourasky Medical Center ethics committee (REC Ref: 0056-20-TLV). Written and verbal informed consent was obtained from all participants after they received a detailed description of the study.

### Participants

Included were patients who developed a major depressive episode as defined by the Diagnostic and Statistical Manual of Mental Disorders – Fifth Edition (DSM-5) [16] up to 12 months after they suffered a stroke or transient ischemic (TIA) and were consecutively recruited from the outpatient clinics at Tel Aviv Sourasky Medical Center. Patients were invited to participate in the study during their routine clinical evaluation after stroke. They were not specifically referred to the Post-stroke Clinic for depressive symptoms. Fifteen patients underwent screening for participation between October 19 2020 to June 1 2021. Ten were medically eligible and consented to the study.

Inclusion criteria were [1] age between 50 and 86 years; [2] diagnosis of stroke/TIA before study entrance and evidence of ischemic infarct on MRI; [3] meet DSM-5 diagnostic criteria for a major depressive episode that developed up to 12 months after the documented stroke; [4] able to sign informed consent, comply with scheduled visits, treatment plan, and other trial procedures.

Exclusion criteria were: [1] hemorrhages and cerebral edema; [2] significant acute medical illnesses that limit the use of Maraviroc, including: severely disturbed liver, kidney or lung function, anemia, hypothyroidism or uncontrolled diabetes; [3] significant acute neurologic illness including: impaired consciousness, Parkinson’s disease, Huntington’s chorea, progressive supranuclear paralysis, brain tumor, subdural hematoma, multiple sclerosis, hydrocephalus, Binswanger’s disease or severe aphasia; [5] dementia or major neurocognitive disorder as defined by DSM-5; [6] history of human immunodeficiency virus (HIV), hepatitis B surface antigen (HBsAg) or hepatitis C antibody (anti-HCV) positive; [7] current or past diagnosis of bipolar or related disorders, intellectual disability, psychotic disorder, schizophrenia, post-traumatic stress disorder (PTSD), and substance use disorders other than nicotine in the past year; [8] intent or plan to attempt suicide in near future; [9] began taking a new antidepressant or anxiolytic agent up to 3 months before enrollment.

### Primary safety endpoint

Any serious treatment-emergent adverse events (TEAEs) TEAEs leading to discontinuation.

### Assessment

Safety evaluations included physical examination, body weight, vital signs, 12-lead electrocardiogram (ECG), clinical laboratory tests, concomitant therapies, and assessment of adverse events’ nature, frequency, and severity (AEs). All AEs, whether volunteered by the patient, discovered by study personnel during questioning, or detected through physical examination, laboratory test, or other means, were recorded in the patient’s medical record and on the appropriate case report forms.

Suicidal ideation and behavior were assessed by a senior psychiatrist, as well as continuous follow-up of mental state and AEs.

AEs were recorded by the following information: The severity grade was used as - mild: usually transient and generally not interfering with normal activities; moderate: sufficiently discomforting to interfere with normal activities; severe: prevents normal activities. The AE outcome (not recovered/not resolved; recovered/ resolved; recovering/resolving, recovered/resolved with sequelae; fatal; or unknown) was recorded.

The presence of a major depressive episode was determined in a structured interview by the study’s senior psychiatrist (OT). Depression severity was assessed using the Montgomery Åsberg Depression Rating Scale (MADRS) [17] and this questionnaire served as the endpoint measure.

We measured self-perception of the overall severity of depressive symptoms using the 16-item quick inventory of depressive symptoms- self-report, QIDS-SR16 [18], a validated depression evaluation tool. Other scales used were the clinical global impression scale (CGI) [19], and the 7-item patient-reported generalized anxiety disorder 7-item scale- GAD-7 [20]. A neuropsychological assessment was based on the NeuroTrax computerized cognitive testing (NeuroTrax Corp., Bellaire, TX), using different versions at each visit.

Changes in depression and anxiety severity and medication side effects were assessed at weeks 2, 4, 7, and 10. Post-treatment assessments were held at weeks 14 and 18.

Changes in functional and behavioral outcomes were assessed using the Stroke impact scale (SIS), the Quality of Life Enjoyment and Satisfaction Questionnaire – Short Form (Q-LES-Q-SF), and the Reintegration to Normal Living Index (RNLI).

See the schedule of all assessments in Supplementary material (Figure S1 and Table S1).

Fasting blood samples were taken with minimal stasis. High sensitivity C-reactive protein (hs-CRP) concentrations in blood were measured using a Boering BN II Nephelometer (DADE Boering, Marburg, Germany).

### Treatment

Participants received an oral (PO) dose of Maraviroc 300 mg once daily in the morning over ten weeks as add-on therapy to their psychiatric pharmacological treatment regimen, which remained stable during the trial.

### Analyses

#### Safety analysis (primary endpoint)

All AEs were coded according to coding dictionaries (MedDRA version 22.0 or higher) and presented in tables by System Organ Class (SOC) and Preferred Term (PT), as well as findings of physical examinations, vital signs, clinical laboratory test results, and concomitant medications.

To evaluate the changes in vital signs and clinical laboratory test results from baseline to the end of treatment at week ten, we used the paired t-test or the Signed rank test (as appropriate) to test the statistical significance of the difference.

#### Efficacy analysis (secondary endpoint)

To evaluate the changes in depression severity during the intervention on the whole sample, data measured at baseline were compared with those at week ten using a paired t-test. After confirming the data were normally distributed, the effect of Maraviroc over time was analyzed with repeated measures ANOVAs, with time being a within-subjects factor and depression scores (as measured by the MADRS) as the dependent variables. Associations between numeric variables were determined using Spearman’s rank correlation analysis (coefficient estimate r).

The response was defined as a ≥ 50% decrease from baseline depression scale scores to the trial endpoint. Significance was evaluated at the α = 0.05 level, two-tailed, after Greenhouse-Geisser corrections. Pairwise comparisons of the study day assessments to baseline for significant main effects and interactions were performed with Bonferroni corrections for multiple comparisons [21].

A sample size of 10 patients was required to obtain sufficient precision for this exploratory trial for safety (see details in Supplemental material). A p-value < 0.05 was considered statistically significant for all analyses. SPSS/WIN (version 29.0, SPSS, Chicago, IL, USA) software was used for all statistical analyses.

## Results

Ten participants with a recent subcortical or cortical stroke, suffering from a major depressive episode were included in the trial. They were administered Maraviroc orally at a daily dose of 300 mg in addition to their current treatment plan. Out of the 10 participants, two were female, and all participants were of Caucasian ethnicity. The average age was 61.5 *±* 7.9 years (range 54–81) and the mean education level was 13.9 *±* 4.2 years. The mean MADRS score at baseline was 30.4 *±* 7.7, pointing to moderate-severe depression. Additional clinical and demographic details of the sample are provided in Table 1. Although we aimed to include participants whose depression developed within a year of their stroke, all participants included developed depression within six months after their documented stroke. The average time from the index stroke to enrollment was 19.4 months (SD: 18.5).

**Table 1:** Demographic characteristics of patients completing the study

Baseline Characteristics	Mean (± SD) or Number (%)
Age, years (SD)	61.5 (7.9)
Sex	
Female, n (%)	2 (20)
Male, n (%)	8 (80)
Current smokers, n (%)	3 (30)
Diabetes mellitus, n (%)	3 (30)
Hypertension, n (%)	10 (100)
Pre-existing depression, n (%)	1 (10)
Education, years (SD)	13.9 (4.2)
Marital Status	
Married, n (%)	8 (80)
Divorced, n (%)	2 (20)
Number of children, n (SD)	2.8 (1.8)
Body-mass index, kg/m^2^ (SD)	28.2 (4.1)
NIHSS at study entrance, median (IQR)	1.5 (0.75-3.0)
Months from index stroke to enrollment (SD)	19.4 (18.5)
Months from development of PSD to enrollment (SD)	15.8 (16.8)
Affected side	
Left, n (%)	5 (50)
Right, n (%)	4 (40)
Bilateral, n (%)	1 (10)
Cortical lesion, n (%)	4 (40)
Individual’s perception of his/her recovery from stroke - on scale from 0-100 (100 = full recovery), median (IQR)	50.0 (32.5–77.5)

IQR, interquartile range; NIHSS, National Institutes of Health Stroke Scale; SD, standard deviation; PSD, post-stroke depression. Entries are mean (SD) or n and %, as indicated

Regarding concomitant antidepressant medication, five patients received monotherapy with an SSRI (4 escitalopram and 1 sertraline), 2 patients received a combination of an SSRI and mirtazapine (1 escitalopram and mirtazapine and 1 fluoxetine and mirtazapine); all patients receiving antidepressants remained symptomatic although receiving treatment. all patients receiving antidepressants remained symptomatic although receiving treatment. Three patients refused the use of antidepressants at the time study entrance due to fear of side effects.

Patients taking concomitant antidepressant medications at the time of screening had been treated for at least 16 weeks before screening. They remained on a stable drug dose for the duration of the study. All included patients completed the ten weeks of study drug treatment; eight completed the post-treatment eight-week follow-up period; two completed only four weeks of follow-up.

### Safety

Maraviroc was well tolerated and no serious adverse events were reported.

There were no changes in weight, liver or renal function, cell blood counts, and electrolytes (Table 2). The adverse effects reported were: 1 dry mouth, 1 overactive bladder, 1 urinary tract infection, 1 leg weakness, 1 dyspnea, 1 dizziness, 1 elevated blood pressure, 1 insomnia. These symptoms were probably unrelated to Maraviroc and resolved before the ten-week treatment period was completed. No serious treatment-emergent adverse events (TEAEs) were reported and no TEAEs led to discontinuation. Pairwise comparison showed no significant difference from baseline to week 10 in systolic and diastolic blood pressure, weight, aspartate aminotransferase (AST), alanine aminotransferase (ALT), bilirubin, creatinine and white blood cells (*p* = 0.104, *p* = 0.521, *p* = 0.876, *p* = 0.211, *p* = 0.970, *p* = 0.950, *p* = 0.313, *p* = 0.586, *p* = 0.100, *p* = 0.078, respectively).

**Table 2:** Mean (*±* SD) for weight (kilograms), vital signs, and liver and renal function on study assessment weeks

	Baseline	Week 2	Week 4	Week 7	Week 10
Weight	80.9 (9.3)	80.9 (9.8)	81 (11.2)	80.8 (10.5)	81.3 (10)
Systolic BP	132.5 (9)	131 (11.3)	130 (18.1)	134.3 (11.3)	130.9 (21.2)
Diastolic BP	81.4 (9.9)	75.4 (10.9)	75.3 (7.1)	81.4 (13.7)	80.3 (11.7)
Pulse	70.5 (11.8)	68.5 (10.4)	72.3 (12)	66.8 (12.9)	69.7 (8.2)
AST, U/L	23.6 (7.8)	23.7 (7.9)	24.1 (8.9)	24.6 (12.2)	23.5 (7.9)
ALT, U/L	25.5 (11.8)	27.5 (9.3)	26.8 (12.7)	27.9 (11.5)	25.4 (11.3)
Bilirubin, mg/dL	0.51 (0.1)	0.47 (0.14)	0.5 (0.12)	0.48 (0.14)	0.48 (0.15)
Creatinine, mg/dL	0.93 (0.24)	0.95 (0.26)	0.98 (0.29)	0.99 (0.31)	0.95 (0.27)

ALT, Alanine aminotransferase; AST, Aspartate aminotransferase; BP, blood pressure; SD, standard deviation

No change was observed in neurological scores (NIHSS) or ECG parameters, as well as suicidal ideation from baseline to week 10. There was no change in weight and no reports of sexual dysfunction from baseline to week 10.

### Effect on depression

**Fig. 1:**
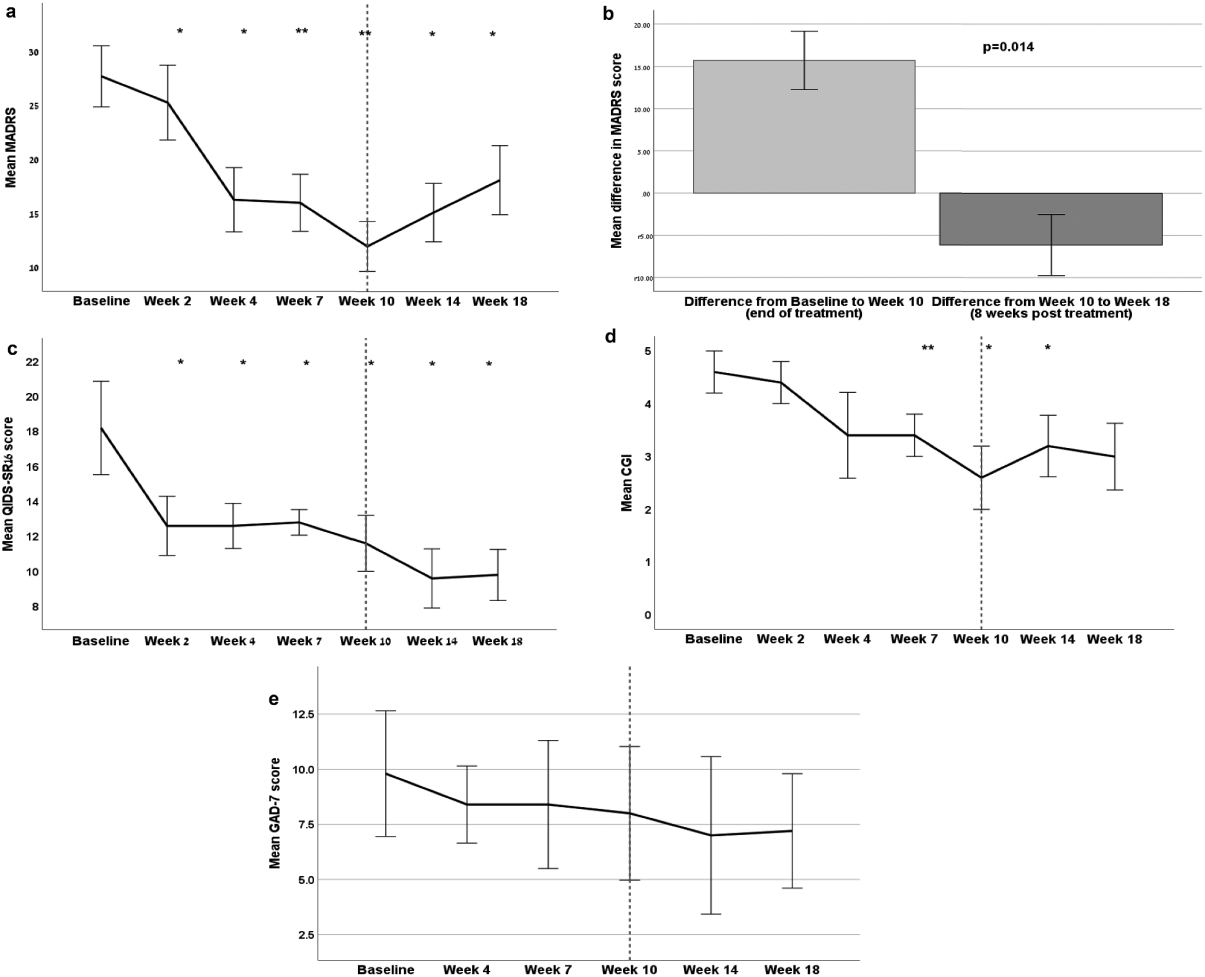
(**A**) Montgomery-Asberg Depression Rating Scale (MADRS) scores of the patients included in the study. **B** Mean difference in Montgomery-Asberg Depression Rating Scale (MADRS) scores over ten weeks of open treatment with Maraviroc versus eight weeks of follow-up. **C** 16-item quick inventory of depressive symptoms- self-report (QIDS-SR16) scores of the patients included in the study. **D** Clinical global impression (CGI) scores of the patients included in the study. **E** GAD-7 scores of the patients included in the study

The mean MADRS score decreased from 30.4 *±* 7.7 at baseline to 14 *±* 8.3 at week 10 (end of treatment period), representing a mean change of -16.4 ± 9.3, t9 = 5.6; *p* < 0.001, (Fig. 1A and B). The effect size of the change in MADRS scores between the baseline and week 10 was substantial, with Cohen’s D value of 1.76 95% and a CI 0.73 to 2.76).

MADRS scores decreased during the study as a function of time (F4,36 = 19.3, *p* > 0.001); pairwise comparison showed a significant difference from baseline starting at week two and continuing through week 10.

Corrected post hoc pairwise comparisons to baseline found that scores at week 4 (mean Δ = 12.8, d = 2.69, 95% CI 2.87 to 22.73; *p* = 0.01); week 7 (mean Δ = 13.3, d = 2.6, 95% CI 3.71 to 22.89; *p* = 0.006); and week 10 (mean Δ = 16.4, d = 2.95, 95% CI 5.54 to 27.27; *p* = 0.003) were significantly lower than baseline (Fig. 1A). Clinical exams by a psychiatrist confirmed significant improvements in depressive symptoms in all completers on week 4, week seven, and week 10 of the trial. After cessation of treatment, this trend reversed and the mean MADRS scores increased from week 10 to week 18 by 6.1 + 9.6 points; *p* = 0.014 (Fig. 1B).

After ten weeks of Maraviroc treatment, 40% (4 out of 10) of patients attained remission, defined as a MADRS total of 10 or less (Fig. 1A), and 50% (5 out of 10) of the patients demonstrated a response, signifying a reduction in MADRS scores by more than 50% from their baseline measurements at week 10.

A non-parametric analysis (utilizing the Mann–Whitney test) to investigate differences in baseline variables revealed that, in comparison to individuals who did not achieve remission, remitters entered the study sooner after their stroke (remitters mean time post-stroke: 8.8 + 5.51 months, while the non-remitters had a mean time post-stroke of 26.5 + 21.2 months; *p* = 0.09, not significant, but may imply a trend). Three of the four remitters were receiving a stable daily dosage of 20 mg escitalopram during the study trial, while one was not taking any antidepressants.

Similar to the observation in the MADRS score, the QIDS-SR16 self-report score also demonstrated a decline from baseline to week 10: -6.8 ± 6.4, t9 = 3.4; *p* = 0.008. Yet, unlike the result observed in the MADRS, the mean QIDS-SR16 score continued to decline slightly through week 18 (after study drug cessation): -7.8 ± 6.6 compared to baseline, t5 = 2.9; *p* = 0.033; Fig. 1C). The Geriatric depression scale (GDS) also demonstrated a decline from baseline to week 10: -3.6 *±* 2.9, *p* = 0.016.

The mean change in the severity of illness using the Clinical Global Impression - Severity (CGI-S) score from baseline to week ten was − 1.7 ± 1.3, t9 = 4.3, *p* = 0.002; Fig. 1D). Mean change in the severity of illness using the Clinical Global Impression - Improvement (CGI-I) score from baseline to week 10: +0.8 ± 0.79, t9 = 3.2; *p* = 0.011).

### Time to response

The mean (*±* SD) time to response for depressive symptoms was 3.1 (*±* 1.2) weeks (*n* = 5, median = 3.5). All patients maintained this response through week 10.

### Effect on anxiety

The mean GAD-7 score at baseline was 8.7 *±* 5.6, and at week ten 7.0 *±* 6.0. The effect size of the difference in anxiety symptoms was not significant (Fig. 1E).

### Effect on cognitive scores

**Fig. 2:**
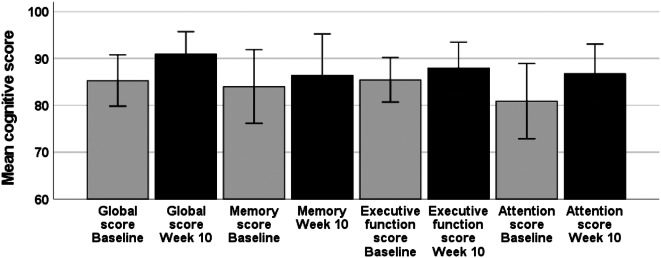
Mean cognitive scores of patients included at baseline and after ten weeks of treatment with Maraviroc

Eight patients improved their cognitive scores from baseline to week 10: the mean global cognitive score for all patients at baseline was 85.3 *±* 14.4, and the mean global cognitive score at week 10 was 90.9 *±* 12.7, *p* = 0.038 (Fig. 2). The mean MoCA score at baseline was 23.7 *±* 1.9, and the mean MoCA score at week 10 was 24.8 + 2.4, *p* = 0.003.

### Effect on function and behavior

Seven patients improved their functional and behavioral scores from baseline to week 10: the mean SIS score for all patients at baseline was 52 *±* 26.5, and the mean SIS score at week 10 was 65.0 *±* 17.3, *p* = 0.014; the mean Q-LES-Q-SF score for all patients at baseline was 46.4 *±* 10.5, and the mean Q-LES-Q-SF score at week 10 was 52.7 *±* 8.7, *p* = 0.003; the mean RNLI score for all patients at baseline was 31.9 *±* 9.3, and the mean RNLI score at week 10 was 36.9 *±* 9.0, *p* = 0.049.

### Effect on C-reactive protein (CRP)

Although within the normal range, mean blood CRP levels decreased significantly from baseline to the end of treatment at week 10 (2.6 *±* 1.9 vs. 1.7 *±* 1.6, *p* = 0.011). Baseline CRP levels correlated with baseline MADRS scores (*r* = 0.762, *p* = 0.017).

## Discussion

This 10-week open-label, proof-of-concept clinical trial is the first to explore the safety and effect of add-on treatment with maraviroc, a CCR5 antagonist, on depressive symptoms in patients suffering from PSD. The main findings were in accordance with our initial hypothesis: [1] Maraviroc was well tolerated, with no documented severe adverse incidents or discontinuations attributed to intolerance [2]. Maraviroc did not affect heart rate, blood pressure, weight, liver or renal function, and cell blood counts [3]. Over the course of the study, we observed an improvement in depressive symptoms as measured by the MADRS: with a mean time-to-response of ~ 3 weeks [4]. Some responders experienced worsening of their depressive symptoms after stopping the study drug. Notably, among the five responders, four achieved remission and not merely an improvement in their depressive symptoms. The common adverse events of infections (upper respiratory and herpes simplex) and postural hypotension, reported in HIV patients, were not observed.

To the best of our knowledge, this is the first time Maraviroc was tested in depressed patients. Our findings agree with prior data implying that blockade of the CCR5 (whether occurring naturally in humans or manually induced in rodents) may improve post-stroke sequela in multiple neuropsychiatric domains [9–11, 22]. We suggest that the two main mechanisms of action that explain the efficacy of CCR5 blockade, as seen in our results, are the modulation of immune processes, including inflammation, and the induction of synaptogenesis.

It is well known that a cerebral infarct can induce a plethora of inflammatory and cytokine signaling, with an upregulation of cellular activity aimed at clearing damaged tissue and restoring severed connections. Previous studies suggest a link between these post-stroke inflammatory responses and the development of PSD [23–27]; Other studies point to left-sided lesions [28], lesion size [29], and extent of small vessel disease in the white matter and subcortical regions as predictors of PSD [30, 31]. Indeed, the biological basis of PSD may differ from that of MDD diagnosed in patients with no known vascular insults. It cannot be examined as a separate entity from the stroke but rather as a process sharing common pathways. This might explain why drugs used to treat “regular” MDD, mainly selective serotonin reuptake inhibitors (SSRIs), often fail to produce an adequate response in patients with PSD - especially with substantial white matter vascular pathology [32, 33]. In a study by Chollet and colleagues, the CCR5-Δ32 mutation had a more pronounced effect on the recovery of neurological impairments in the NIH stroke scale than fluoxetine [34]. In our small study, CRP levels, which showed a correlation with depressive scores, decreased significantly from baseline to the end of treatment at week 10, implying an anti-inflammatory effect. We have previously reported that carriers of the CCR5-Δ32 mutation had lower C-reactive protein (CRP) at admission to hospital after stroke [11].

In the primate brain, CCR5 blockade attenuated activation of glial cells and neuroinflammation [35]. In humans, Maraviroc inhibited chemokine-dependent migration of inflammatory cells through the endothelium [36]. Thus, the potential anti-inflammatory effect attributed to CCR5 blockade may be at least partially responsible for alleviating post-stroke depression symptoms.

There is a growing consensus that ischemic lesions (single and multiple) of the neural circuits that connect the prefrontal cortex, basal ganglia, thalamus, and amygdala may disrupt mood regulation and executive function, leading to depression. Furthermore, there appears to be a threshold by which further damage to specific white matter tracts triggers the onset of PSD [37]. Enabling recovery of affected brain regions is therefore crucial. As previously mentioned, CCR5 kd in rodents in an experimental stroke model was shown to prevent the loss of synaptic connections in the adjacent cortex, promote the formation of new connections, and stabilize dendritic spines during maximal spine loss. CCR5 blockade induced a remarkable degree of axonal sprouting in the bi-hemispheric or callosal connections of the pre-motor cortex ^10^. In humans, recovery after stroke is associated with plasticity in the pre-motor cortex and is partly driven by bilateral connections [38]. It is prudent to assume that the same process mediates recovery from PSD – an assumption strengthened by our previous finding that the CCR5-Δ32 mutation is a possible protective factor for PSD [11].

Our study has several limitations. First, it is a small open-label pilot study powered for safety only and not powered for efficacy. The results of such a pilot study could be used to rank-order Maraviroc as a potential therapy thereby proving an empirically justified approach to therapy development. Second, we cannot exclude a significant placebo effect because this was an open-label design. Therefore, all the efficacy data should be considered as hypothesis-generating, and larger placebo-controlled trials should be conducted to verify these results. Two such studies are underway by our group: The MARCH trial testing maraviroc for post-stroke cognitive impairment [39], which will also examine PSD as a secondary endpoint, and another large placebo-controlled study that will test maraviroc in PSD patients (NCT04966429). Third, in the current study, the interval between the index stroke and study entry varied significantly. This makes the interpretation of our results more challenging. Lastly, although the loss to follow-up was mild, extrapolation was necessary to estimate probable measures in patients lost to follow-up.

Our study’s strengths included its novelty, the inclusion of patients not responding adequately to antidepressant medication, and the rigorous follow-up of patients using psychiatric and neurological (cognitive) evaluations.

Despite its limitations, this open-label proof-of-concept trial suggests that daily Maraviroc augmentation treatment is safe in PSD patients. It may also potentially improve PSD symptoms with few adverse events. This pilot study is an important step in designing a larger, more comprehensive randomized-control study of Maraviroc in PSD.

### Electronic supplementary material

Below is the link to the electronic supplementary material.

Supplementary Material 1

## Data Availability

The datasets generated during and/or analyzed during the current study are available from the corresponding author on reasonable request.

## Declarations

### Consort guidelines

Our study adheres to CONSORT guidelines.
