# Supplementary material for: Blocking CCR5 activity by maraviroc augmentation in post-stroke depression: a proof-of-concept clinical trial
Source: BMC Neurol. 2024 Jun 6;24:190. doi: 10.1186/s12883-024-03683-3 (PMC11155100; doi:10.1186/s12883-024-03683-3)
Supplement: Supplementary file 1 — Supplementary Material 1 [file 12883_2024_3683_MOESM1_ESM.docx]

**Supplementary Figure S1: Flow diagram summarizing the process of the trial**

Identify patient with post-stroke depression

Check eligibility

Consent

**Collect baseline data**: physical exam, vital signs, computerized cognitive tests, questionnaires, psychiatric assessment, blood samples.

Start treatment with 300 mg/day Maraviroc

**Follow-up assessment on week 2 and weeks 4, 7, and 10:** physical exam, vital signs, computerized cognitive tests (at week 10), questionnaires, psychiatric assessment, record of AEs related or not related to the medication assignment, concomitant medications, pill counts, blood samples.

**Telephone contact assessment on weeks 3, 5, 8, and 9:** record of AEs, and concomitant medications.

**Follow-up assessment on weeks 14, and 18 (post-treatment assessments):** physical exam, vital signs, questionnaires, psychiatric assessment.

Supplemental Methods: Sample size calculation

The percentage of pilot study participants who tolerate a new treatment or the proportion of eligible patients who provide informed consent for participation would be representative of the tolerance or participation rates if all potential participants were studied. Providing a means to estimate these quantities enables early indicators of treatment tolerability and enrollment feasibility.

Weight gain and sexual dysfunction are well documented with the current anti-depressants, usually selective serotonin reuptake inhibitors (SSRIs) or Serotonin-Norepinephrine Reuptake Inhibitors (SNRIs). Another side effect of SSRIs is hyponatremia, which can limit their use in stroke patients.

It is of clinical interest to identify augmentative therapies that can alleviate some of the known side effects affecting a high percentage of patients receiving SSRIs or SNRIs (an average of 30% of people treated with an SSRI reported sexual dysfunction^1^, while 25% of individuals had a significant body weight increase^2^ and an average of 22% had hyponatremia^3^). Therefore, it is desirable to rule out potential treatments not consistent with this rate of sexual dysfunction, weight gain, and hyponatremia before considering more conclusive testing. Using the following equation,

**
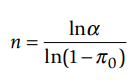
**

(where π is the probability of the adverse event of interest)

we would conclude that the augmentative therapy has sexual dysfunction rates less than 0.31, at the 90% confidence level, if a total of 10 patients are recruited and all 10 do not experience sexual dysfunction. We would conclude that the augmentative therapy has weight gain rates less than 0.25, at the 90% confidence level, if a total of 8 patients are recruited and all 8 do not experience weight gain. We would conclude that the augmentative therapy has hyponatremia rates less than 0.22, at the 90% confidence level, if a total of 9 patients are recruited and all 9 do not experience weight gain. Therefore, an initial sample size of 10 patients would be sufficient to identify augmentative therapies, such as Maraviroc. The results of such a pilot study could then be used to rank-order Maraviroc as a potential therapy thereby proving an empirically justified approach to therapy development.

1. Higgins A, Nash M, Lynch AM. Antidepressant-associated sexual dysfunction: impact, effects, and treatment. Drug Healthc Patient Saf. 2010;2:141-50.
2. Gafoor R, Booth HP, Gulliford MC. Antidepressant utilisation and incidence of weight gain during 10 years' follow-up: population based cohort study. BMJ. 2018 May 23;361:k1951.
3. Egger C, Muehlbacher M, Nickel M, Geretsegger C, Stuppaeck C. A review on hyponatremia associated with SSRIs, reboxetine and venlafaxine. Int J Psychiatry Clin Pract. 2006;10(1):17-26.

**Supplemental Protocol**

**Title:** **Safety of Maraviroc for post-stroke depression**

**Introduction:**

# Posts-stroke depression (PSD) is the most frequent neuropsychiatric sequela of stroke, with a prevalence rate of 18 to 61% (a 3-fold increase when compared with the general population)^1,2^. PSD is associated with increased mortality, higher disability, lower quality of life and a greater risk for cognitive decline^3,4,5^. Despite significant clinical relevance, the relationship between stroke and depression, assumed to be multifactorial, remains poorly understood, and specific mechanism-based treatment targets are lacking.

Currently there are no "gold standard" therapies for PSD or post stroke anxiety symptoms. Therapeutic options do not differ from those recommended to the general population, but controlled clinical trials examining their efficacy in this specific sub group of patients demonstrated conflicting results, and two recent randomized control trials using fluoxetine and escitalopram (both specific serotonin reuptake inhibitors – SSRI's) in this population showed no significant efficacy^6,7^.

# Although multiple treatments for Major depressive disorder (MDD) are available, nearly 30 to 50% of patients do not fully remit from the disorder using current biogenic amine-based antidepressant drugs^8,9^. Limitations of currently available antidepressants include delayed onset of therapeutic action, troubling side effects and low remission rates after multiple courses of pharmacotherapy. Indeed, the large-scale Sequenced Treatment Alternatives to Relieve Depression trial (STAR*D) demonstrated that for 70% of patients, a single treatment trial with a SSRI did not bring upon remission^10^. After 2 sequential treatment interventions remission rates were only 50% to 55%, and the probability of achieving remission with third- and fourth-step therapy options was considerably lower^11^.

Elderly patients and stroke survivors are especially susceptible to side effects that can limit the use of SSRI's, such as hyponatremia. In addition, meta-analyses demonstrate that SSRI's have little to no effect on cognitive function^12^, an important component of post-stroke recovery, with much influence on emotional sequelae.

# Accumulating evidence suggests that inflammatory processes and neural-immune interactions are involved in PSD. Related theories focus on synaptic plasticity as an important means of recovery from PSD, after converged molecular and cellular mechanisms including inflammation cause atrophy of neurons, loss of glutamatergic synaptic connections and dysfunction of the circuitry that is essential for mood regulation and cognitive function^13,14^. Indeed, PSD can be perceived as a psychoneuroimmunological disorder in which inflammatory mechanisms involving cytokines and chemokines play a crucial role^15,16^. Both acute stroke patients and depressed patients consistently show higher levels of pro-inflammatory cytokines, acute phase proteins, chemokines and cellular adhesion molecules^17,18.^.This suggests that psychotropic drugs that have central anti-inflammatory properties may serve as a mechanism based treatment option for depression, thus providing a new mode of treatment..

**The C-C chemokine receptor type 5 (CCR5),** is known to be involved in immune processes and neuroplasticity, and is highly expressed in T cells and macrophages, as well as microglia, astrocytes and neurons in multiple brain regions. CCR5 is a pro-inflammatory receptor and inhibition of its signaling has been shown to enhance plasticity processes in hippocampal and cortical circuits^19^. Other reports show that CCR5 blockade attenuated activation of glial cells, maintained the integrity of endothelial monolayer, reduced the infiltration of T cells, and attenuated neuroinflammation^20^.

We have recently tested the effects of the naturally occurring CCR5-Δ32 mutation in 435 post-stroke patients (the TABASCO prospective cohort). In this cohort, about 15% of patients, mostly Ashkenazi Jewish individuals, were carriers of the mutation. This group showed significantly better cognitive and functional outcome 2 years post-stroke^20^. Unpublished data further demonstrated that this sub group of patients had also significantly less depressive and post-traumatic symptoms, 6 and 12 and 24 months after the index event, implying that impaired activity of the CCR5 may have therapeutic advantages in both stroke cognitive recovery and depressive symptoms alleviation. Our findings suggest that the anti-depressive effect of the mutation might even be greater than its "cognitive protection" effect. Interestingly, Ogłodek et al. in her studies found a remarkable rise in the level of CCR5 and its chemokines in serum from patients with depression and post-traumatic stress disorder (PTSD)^21-23^.

On this basis, we suggest repurposing **Maraviroc** (Selzentry), the Food and Drug Administration (FDA) approved CCR5 antagonist for R5-tropic HIV-1 infected patients, as a new mechanism-based treatment option for PSD.

Together with our colleagues, we have recently shown^20^ that Maraviroc modulates several parallel signaling cascades implicated in learning, memory and in depression, including the suppression of adenyl cyclase ^,24,25^, as well as the activation of the PI3K/AKT and P44/42 MAPK signaling^26^. These findings support the application of brain permeable CCR5 antagonists, not only as a combination drug in antiretroviral therapy, but also as a treatment for cognitive deficits and depression caused by HIV. We offer using this same drug, that mimics the action of the CCR-5 mutation as novel treatment option for PSD.

# Toxicology

Maraviroc is the only CCR5 antagonist currently approved by the FDA, the European Commission and the Health Canada for treatment of patients infected with R5-tropic HIV-1. The drug is a small molecule metabolized by CYP3A4, with a good pharmacokinetic profile, relatively low protein binding and high bioavailability when given at standard doses twice a day. Maraviroc appears to be well tolerated, but the dose has to be adjusted when given with CYP3A4 inducers or inhibitors, primarily drugs that are also used for HIV therapy, but also for several anticonvulsants. Patients who take these medications will be excluded from the proposed trial. The drug should be used cautiously in patients with a history of orthostatic hypotension and patients with pre-existing liver dysfunction or co-infection with hepatitis B or C (these patients will also be excluded from the proposed trial). Maraviroc does not appear to cause clinically significant changes in concentrations of other medications. It is moderately lipophilic, so it can penetrate the blood-brain barrier. At a single dose of 150 mg or 300 mg, time to maximum concentration occurred by 2 hours post-treatment in humans. The terminal half-life is 14-18 hours, so a single dose used in the proposed protocol should be adequate, rather than the twice-daily (BID) treatment for AIDS.

**Based on Pfizer’s reports on premarketing and post marketing studies of Maraviroc and review of the literature, no dose adjustment is necessary even in patients with mild-to-moderate renal impairment. The drug does not affect the QT interval.** **At high doses (600 mg or more), it may induce orthostatic hypotension, so it is recommended that users who also take antihypertensive medications be asked about symptoms of orthostatic hypotension.** Of note, 8% of patients in active and placebo drug groups described orthostatic symptoms in a large trial. In HIV trials, 1.3% of subjects had cardiovascular events, more than in the placebo group, but the link to the drug was unclear and symptoms occurred only in those with known cardiac disease. Also, no greater incidence of infection, rash or other CNS symptoms were noted in these patients. **An occasional Stevens-Johnson syndrome and drug rash with eosinophilia and systemic symptoms did occur (seen only in postmarketing surveillance, not in controlled trials), so complaints of rash, fever, joint or muscle aches, blisters, facial edema etc. will be part of our weekly phone call surveillance plan. Participants will be told to stop their medication immediately should such symptoms occur, and their physician will be notified.** Of note, St John’s wort should not be used with Maraviroc, since it decreases the concentration of the medication.

**Maraviroc is metabolized by the liver, so using it in persons with more than mild hepatic disease, especially in the presence of a CYP3A inhibitor, would have to be closely monitored; we will therefore not include these patients in this study.**

Clinical aspects

**Pharmacokinetics**: Maraviroc pharmacokinetics was studied in 28 phase I/IIa studies (complete profiles) and 3 phase IIb/III studies (with sparse sampling). The evaluation was performed after single-dose intravenous administration (1–30 mg) as well as oral single-dose (1–1200 mg) and multiple-dose administration (3–900 mg twice daily [BID] and 1200 mg once daily [QD]). The following formulations were used during development: powder for oral solution in phase I, 5 mg/25 mg/50 mg/100 mg/150 mg tablets in phase I/IIa and 150 mg in phase IIb/III. An intravenous formulation was used to determine absolute bioavailability. The commercial formulations (150 mg, 300 mg) were not used in the clinical trials. The analytical methods used to analyze Maraviroc have been adequately validated.

**Absorption**: The absorption of Maraviroc is highly variable with multiple peaks. The mean Tmax was between 2 and 3 hours with individual values ranging from 0.5 to 8 hours (with food). The absolute bioavailability for Maraviroc was 23% at 100 mg, and it has a predicted bioavailability of 31% at 300 mg. The absorption of Maraviroc is dose dependent, likely attributed to saturated efflux transporters in the intestine. Maraviroc is highly soluble in aqueous media across pH 1–7.5, has an efflux ratio >10 in Caco-2 cell monolayers and is a substrate for P-gp and the multidrug resistance protein.

**Bioequivalence:** The commercial tablet formulations (150 mg, 300 mg) are completely dissolved within 30 minutes (>90% within 15 minutes), and hence, dissolution will not be rate limiting for the absorption of Maraviroc. Bioequivalence was shown between the commercial 300 mg tablet and research formulation (2 x 150 mg). The solution had a 12% higher bioavailability than the research tablet.

**Metabolism**: The metabolism of Maraviroc was evaluated in 3 healthy male subjects after administration of 300 mg 14C Maraviroc as an oral solution in a fasted state. Whole blood samples and plasma samples were collected on days 1 to 6 at specified times up to 120 hours post-dose to measure plasma Maraviroc and metabolite UK-463,977 concentrations and radioactivity and for metabolite profiling, respectively. Urine and feces were collected to measure urinary and fecal radioactivity and for metabolite profiling up to at least 120 hours post-dose on day 1. UK-463,977 concentrations were also determined in urine. Unchanged Maraviroc was the main circulating component in plasma (42% of plasma radioactivity), and the metabolites UK-408,027 (22%), an amine analogue (11%) and UK-463,977 were also identified in plasma. The metabolites UK-408,027 or UK463,977 appear not to accumulate with time.

Maraviroc achieves concentrations within the EC90 range in CSF and showed viral suppression in CSF. The CSF:plasma ratio of Maraviroc was reported as 0.03 (0.01–0.10) ^27,28^.

**Hepatic impairment**: A study involving patients with mild and moderate hepatic impairment (Child-Pugh A and B) as well as individuals with normal hepatic function has been conducted^29^. Administration of Maraviroc (300 mg single dose) to patients with mild and moderate hepatic impairment resulted in mean values of area under the curve (AUC) last that were 25% and 45% higher, respectively, than in individuals with normal hepatic function (geometric means and corresponding 90% confidence intervals [CI] for the comparisons were 125% [84.7%, 185%] and 145% [100%, 212%]). Smaller differences in Cmax were noted with mean values 11% and 32% higher for mild and moderate impairment compared to normal function, respectively. As expected, mean CL/F decreased with increasing hepatic impairment, although the differences between mild hepatic impairment and normal hepatic function were minimal. Mean renal clearance (CLR) was higher in individuals with moderate hepatic impairment compared to those with normal hepatic function. The mechanism for this increase in CLR is not known. Mean Tmax and t1/2 did not appear to be affected by hepatic impairment. The data are limited with wide confidence intervals for the comparisons to individuals with normal hepatic function.

**Renal impairment**: Studies in patients with renal impairment have not been performed. In patients without concomitant administration of CYP3A4 inhibitors, renal excretion constitutes a minor elimination pathway (about 23% of total clearance). In these patients, decreased renal function will likely have a limited effect on Maraviroc exposure. In patients with concomitant administration of CYP3A4 inhibitors, e.g., protease inhibitors, renal clearance will constitute up to ~ 70% of total clearance.

Clinical safety

Phase 1 single- and multiple-dose studies in healthy volunteers, conducted in 2001 and the first half of 2002, demonstrated that Maraviroc was safe and well-tolerated in multiple doses up to 300 mg BID, that it had a pharmacokinetic profile compatible with QD or BID oral dosing, that it could be combined with other antiretrovirals and that doses ≥100 mg BID resulted in exposure above the geometric mean antiviral IC90 in vitro.^30,31^

**Patient exposure in phase II studies:** 595 healthy patients and 37 patients with HIV have been exposed to Maraviroc in doses ranging from 1–1200 mg. In 2 multiple dose-finding phase II studies, 66 patients with HIV were exposed to Maraviroc (25–300 mg) for 10 days. Long-term safety data (minimum 24 weeks) were obtained in the main and supportive studies. In addition to the 3 previously presented studies in treatment-experienced patients (A4001027, A4001028 and A4001029), supportive safety data (*n* = 174) were provided from an ongoing study in treatment-naïve patients (A4001026). In this study, a Maraviroc treatment arm (300 mg QD) was stopped due to an increased incidence of treatment failure, and Maraviroc 300 mg BID open-label was offered. A total of 964 treatment-experienced patients received at least 1 dose of Maraviroc, including 840 CCR5-positive patients in the 2 pivotal studies. In the 2 pivotal trials (A4001027, A4001028), patients were exposed to Maraviroc for a median of 8 months); the total exposure (580 patient years) was around five-fold that of placebo exposure (124 patient years).

**Adverse events (AEs):** AEs were similar in frequency and character in patients treated with Maraviroc or placebo and were expected in this treatment population. Furthermore, no relevant differences in AEs (including serious AE) were seen in Maraviroc given QD versus BID. Infections (upper respiratory and herpes simplex) were somewhat more common with Maraviroc than with placebo, also after adjustment of exposure. Herpes simplex as a manifestation of immune response inflammatory syndrome (IRIS) is a well-known phenomenon and could be one possible explanation for this particular finding. AIDS-related infections and malignancies were not more common with Maraviroc, and autoimmune disorders were not reported. No major safety concerns were found with Maraviroc as part of the antiretroviral regimen in treatment-experienced patients. The dose-limiting adverse event, postural hypotension, appeared to be clinically manageable with the chosen dosage of 300 mg. Maraviroc was well tolerated, with the same frequency of study-drug discontinuation for Maraviroc and placebo. The spectrum of AEs reported, including serious AEs and deaths, did not reveal any specific issues considering the population studied. The frequency of liver-related AEs does not raise any concerns for liver toxicity. In late 2004, four large studies were initiated (phase IIb and phase 3), with 4794 patients screened at more than 200 sites in the United States, Canada, Europe, Australia, South Africa, Mexico and Argentina. An in-depth review of all data for evidence of hepatotoxicity for MVC and a high level of vigilance for any signals did not find any evidence for a systematic increase in hepatic enzymes or other markers for hepatotoxicity. Shortly afterward, concerns were raised regarding a potential increased risk for certain malignancies, and initially, there were concerns that this could be a class effect based on the immune-modulatory potential of CCR5 antagonists, but review of data from other vicriviroc studies, as well as the MVC studies, did not support this theory.^32^

Maraviroc (150 mg and 300 mg BID) received approval for use in the United States in August 2007 and in the European Union in September 2007.

# A large, open-label safety study of Maraviroc was conducted at 262 sites worldwide in 1032 R5 HIV-positive, treatment-experienced patients. The data demonstrated that Maraviroc was well tolerated alone and in combination with other antiretroviral medications.^33^ Despite concerns regarding the hepatic safety of CCR5 antagonists, an extensive data analysis did not show a significant difference in severe hepatotoxic effects between Maraviroc and placebo.^34,35^

Design:

# We plan to perform a proof-of-concept open label add-on clinical trial of 300 mg Maraviroc given once daily for 10 weeks in 10 patients with PSD. Depression scores over time will serve as outcome measures and each patient function will be compared before and after treatment with study drug, beyond current treatment with other anti-depressants, such that the efficacy will be analyzed only beyond the efficacy of current treatments. The study will consist of 3 phases: (1) screening, (2) open-label treatment (weeks 1-10), and (3) post-treatment follow-up (8 weeks). The primary objective will be safety and tolerability of Maraviroc 300 mg per day in subjects with PSD. If proved safe, we plan continued research to establish efficacy using different dosages of Maraviroc

Objectives

# This study will evaluate the safety and efficacy of Maraviroc 300 mg per day in patients suffering from PSD.

Primary objective:

1. To investigate the safety and tolerability of Maraviroc 300 mg per day in subjects with PSD.

Secondary Objectives:

1. To investigate the safety and tolerability of Maraviroc 300 mg in PSD subjects, with special attention to: effects on heart rate and blood pressure.
2. To evaluate the efficacy of Maraviroc 300 mg per day in improving depressive symptoms in subjects with PSD, as assessed by a change from baseline in the Montgomery-Asberg Depression Rating Scale (MADRS) total score and in improving cognitive, functional, and behavioral outcomes.

To evaluate the effect of treatment with Maraviroc 300 mg per day for 10 weeks on blood inflammatory markers in subjects with PSD.

3. To assess the effect of Maraviroc 300 mg in PSD subjects on: a. Depressive symptoms, as assessed by the 16-item Quick Inventory of Depressive Symptomatology- Self Report (QIDS-SR16) b. Remission, defined as a MADRS total score ≤10 c. Response, defined as a ≥50% reduction from baseline in MADRS total score d. The severity of illness using the Clinical Global Impression - Severity (CGI-S) and the Patient Global Impression - Severity (PGI-S) e. Symptoms of anxiety as assessed by the Generalized Anxiety Disorder 7-item Scale (GAD-7). d. Cognitive status, as assessed by cognitive score based on repeatable computerized battery of cognitive tests (Neurotrax) and Montreal Cognitive Assessment (MoCA). e. Functional outcome, as assessed by the stroke impact scale (SIS), the activities of daily living (ADL) score, and Reintegration to Normal Living Index (RNL).

Methods

We plan to enroll ten adults with PSD.

### Interventions

Participants will be outpatients diagnosed with PSD up to 12 months after the index event. Following baseline assessments, all participants will receive the active treatment - Maraviroc 300 mg once daily for 10 weeks in an open-label design. They will be followed for another 8 weeks after cessation of Maraviroc. The study intervention will be given as add-on therapy to the patient’s usual prescribed care (treatment as usual),thus all participants will continue their existing antidepressant treatment without change. All participants will be instructed to continue their usual treatment for the whole trial duration. Endpoints:

Primary Safety Endpoint: No serious treatment-emergent adverse events (TEAEs), no TEAEs leading to discontinuation.

Primary Efficacy Endpoint: The primary efficacy evaluation will be the MADRS total score as measured by the change from baseline (i.e., Day 1 pre-dose) to Week 10.

Secondary Endpoints: • Proportion of responders • Proportion of subjects in remission • Change from baseline to week 10 in subject-reported depressive symptoms using the QIDS-SR16. • Change from baseline to week 10 in severity of illness using the CGI-S and CGI-I. • Change from baseline to week 10 in severity of illness using the PGI-S and PGI-C. • Change from baseline to week 10 in anxiety symptoms, as measured by the GAD-7. • Change from baseline to week 10 in the SIS, ADL, and RNL scores.

Neuroimaging acquisition and processing A magnet resonance imaging (MRI) protocol will be performed before visit 1 and will consist of previously described pulse sequences ^36^. All axial slices will be prescribed on the same orientation, covering the whole brain, aligned along the fourth ventricle-orbitofrontal orientation. MRI analyses will include assessment of white matter (WM) hyperintensities, tissue segmentation and Brain atrophy measures as we have previously described^36^.

**Characteristics of human subjects**

**Inclusion Criteria**

1. Men and women aged 50 to 86 years;
2. Able to fully comprehend and sign an informed consent form;
3. Diagnosis of stroke/TIA prior to study entrance and evidence of ischemic infarct and/or lacunar infarcts on MRI (defined as sharply demarcated hypointense lesions <20mm on T1-weighted images with corresponding hypointense lesions with hyperintense rim on FLAIR) and/or deep Cerebral Microbleeds (CMB) (defined as round hypointense lesions on T2-weighted gradient echo-images with a diameter < 10 mm in neuroimaging.
4. Meet Diagnostic and Statistical Manual of Mental Disorders – Fifth Edition (DSM-5) diagnostic criteria for major depressive episode that developed up to 12 months after the documented stroke/TIA.
5. Able to comply with scheduled visits, treatment plan, and other trial procedures;
6. Treatment with other antidepressants is allowed but subjects will be asked not to change their regular treatment regime during the trial.

**Exclusion Criteria:**

1. Hemorrhages and cerebral edema (e.g., subarachnoid haemorrhage, intracerebral hemorrhage, subdural hematoma, epidural hematoma)
2. Patients in a state of coma or with severe disturbance of consciousness, aphasia, agnosia, or deafness that subsequently affects expression and communication.
3. Significant acute medical illness including: drug overdose, severely disturbed liver, kidney or lung function, anemia, hypothyroidism, or uncontrolled diabetes.
4. Significant acute neurologic illness including: impaired consciousness, Parkinson's disease, Huntington's chorea, progressive supranuclear paralysis, brain tumor, subdural hematoma, multiple sclerosis, hydrocephalus, Binswanger's disease, or severe aphasia
5. Patients diagnosed with dementia or major neurocognitive disorder as defined by DSM-5at screening, or other neurological conditions that might dominate the clinical picture (multiple sclerosis, Parkinson's disease, epilepsy, Huntington's chorea, progressive supranuclear paralysis, brain tumor, subdural hematoma, multiple sclerosis, hydrocephalus, Binswanger's disease, etc.)
6. Subject has a history of human immunodeficiency virus (HIV), hepatitis B surface antigen (HBsAg) or hepatitis C antibody (anti-HCV) positive, or other clinically active liver disease, or elevated hepatic transaminases or bilirubin, or tests positive for HIV at Screening.
7. History of renal insufficiency or serum creatinine over 1.6;
8. Subject has a current or past diagnosis of bipolar or related disorders, intellectual disability, cluster B personality disorder (e.g., borderline personality disorder, antisocial personality disorder, histrionic personality disorder, and narcissistic personality disorder), psychotic disorder, schizophrenia, PTSD , and substance/alcohol use disorders other than nicotine in the past year (including barbiturates, methadone, opiates, cocaine, cannabinoids, and amphetamine/ methamphetamine).
9. Subject has suicidal ideation with intent to act during the screening phase or on Day 1 per investigator’s clinical judgment, or has a history of suicidal behavior within the past year; or subject has homicidal ideation/intent at Screening or on Day 1.
10. Subject has a history of malignancy within 5 years before screening (exceptions are squamous and basal cell carcinomas of the skin and carcinoma in situ of the cervix, or malignancy that in the opinion of the investigator is considered cured with minimal risk of recurrence).
11. Subject has known allergies, hypersensitivity, intolerance, or contraindication to Maraviroc or its excipients.
12. Subject has received an investigational drug (including investigational vaccines) or used an invasive investigational medical device within 60 days before the planned first dose of study drug or is currently enrolled in an investigational study.
13. Subject is a woman who is pregnant, breast-feeding, or planning to become pregnant while enrolled in this study or within 3 months after the last dose of study drug.
14. Subject has any condition for which, in the opinion of the investigator, participation would not be in the best interest of the subject (e.g., compromise the well-being) or that could prevent, limit, or confound the protocol-specified assessments.
15. Subject has had major surgery, (e.g., requiring general anesthesia) within 2 weeks before screening, or will not have fully recovered from surgery, or has surgery planned during the time the subject is expected to participate in the study.
16. Use of drugs with possible interactions with Maraviroc.

**Prohibitions and Restrictions**

- Potent CYP3A4 inhibitors are not permitted within 1 week or within a period less than 5 times the drug’s half-life, whichever is longer, before the first dose of study medication and throughout the study.
- Potent CYP3A4 inducers are not permitted for 30 days prior to the first dose of study medication and throughout the study.

Safety Evaluations

Physical examination, body weight, vital signs, 12-lead ECG, clinical laboratory tests, and evaluation of adverse events and concomitant therapies will be performed throughout the study to monitor subject safety. Suicidal ideation and behavior will be assessed by a psychiatrist, as well as continuous follow-up of mental state and adverse events after cessation of Maraviroc treatment.

Primary Efficacy Measures:

**MADRS** -The 10-item clinician-administered MADRS was designed to be used in subjects with MDD to measure the overall severity of depressive symptoms (Montgomery and Asberg 1979). The MADRS has been validated, is reliable, and is acceptable to regulatory health authorities as a primary scale to determine efficacy in major depression. The structured interview guide for the Montgomery Asberg Depression Rating Scale (SIGMA) will be used for each administration (Williams 2008). Using structured interview guides have previously been shown to increase the reliability of given scales. In depression, ‘response’ is commonly defined as a ≥50% reduction in the initial symptom score and remission is typically defined as a total score of ≤10 (Montgomery 1994). The primary efficacy evaluation will be the change from baseline in the MADRS total score in the treatment phase.

Secondary Efficacy measures:

**QIDS-SR16** - The patient administered 16-item quick inventory of depressive symptoms- self report (QIDS-SR16) is designed to be used in patients with MDD to measure the overall severity of depressive symptoms (Rush 2003; Trivedi 2004).

**CGI-I and CGI-S** - The clinical global impression – improvement (CGI-I) is a 7 point scale that requires the [clinician](https://en.wikipedia.org/wiki/Clinician) to assess how much the patient's illness has improved or worsened relative to a baseline state at the beginning of the intervention; The clinical global impression – severity (CGI-S) evaluates the severity of psychopathology from 1 to 7, and will provide an overall clinician-determined summary measure that takes into account all available information, including knowledge of the subject’s history, psychosocial circumstances, symptoms, behavior, and the impact of the symptoms on the subject’s ability to function (Guy 1976).

**GAD-7** - The 7-item patient-reported generalized anxiety disorder 7-item scale (GAD-7) is a brief and valid measure of overall anxiety (Spitzer 2006). Each item is rated on a 4-point scale (0 to 3), with the total score range from 0-21 (higher scores indicating more anxiety).

**GDS** – Geriatric depression scale, that has been has been tested and used extensively with older populations.

**PGI-S and PGI-C** - Patient Global Impression scales are commonly used measures of symptom severity, treatment response and the efficacy of treatments. The patient global impression - severity (PGI-S) will provide an overall patient-rated summary measure that assesses the severity of the subject’s MDD. The patient global impression of change (PGI-C) will provide an overall patient-rated summary that assesses subject perception of change in their MDD since starting study treatment (Rush 2005).

.

**MoCA** - Montreal Cognitive Assessment.

**SIS** - stroke impact scale.

**ADL** - the activities of daily living score.

**RNL** - Reintegration to Normal Living Index.

**Overview of Study Design**

This is a proof-of-concept, open-label, single center study conducted in 10 male and female adult subjects with PSD. Each subject will participate in 3 phases: (1) a screening phase of up to 1 week, (2) an open-label treatment phase of 10 weeks, and (3) an 8-week post-treatment (follow up) phase. The end of study will occur when the last subject in the trial completes his/her last study assessment.

Screening and Baseline assessments

After giving informed consent, subjects will be screened to determine eligibility for study participation. Note: Subjects who are not currently taking an antidepressant at screening are eligible to participate in this study (i.e., subjects may participate in the study whether or not they are taking an antidepressant). Subjects who are taking an antidepressant at the screening visit must have been receiving a stable dose (i.e., dose has been unchanged for at least 2 months before the screening visit) and will be guided to continue the antidepressant at the same dose during the treatment phase. All adverse events and special reporting situations, whether serious or non-serious, will be reported from the time a signed and dated informed consent form (ICF) is obtained until completion of the subject's last study-related procedure (which may include contact for follow-up of safety). If a subject withdraws before the end of the treatment phase for reasons other than withdrawal of consent, the early termination visit should be conducted at the time of discontinuation, followed by completion of the posttreatment phase. All subjects will self-administer the study medication.

**Recruitment and retention plans**

Screening and patient evaluation:

Participants will be out-patients referred from the community or the hospital’s ambulatory services, who meet entrance and exclusion criteria. After receiving a detailed explanation and signing informed consent, the participant will be further screened. All identifying information of participants will be saved in a separate password protected study log available only to study investigators. All the gathered data will be de-identified and coded with a pre-assigned study ID.

Eligible patients enrolled in the study will undergo a baseline visit (Week 1), in which they will receive the study drug following completion of all relevant assessments. During the first administration, participants will remain under clinical supervision and vital signs monitoring for 2 h at the Tel Aviv Sourasky Medical Center. Blood pressure and heart rate will be recorded before discharge. Patients will be evaluated every 2 weeks during the trial using multiple depressive, anxiety and functional questionnaires as well as cognitive tests. A final safety and efficacy assessment will be held 8 weeks after the patient’s last dose (Week 18).

Open-label treatment period:

On Day 1, subjects will start the study drug. A 1-week supply of capsules will be handed to participants after the consent has been signed, and baseline measurements obtained, usually on the same day. The second 2-weeks supply will be provided during a scheduled interim measurement assessment at TLVMC near the end of the 2nd week on medication. Participants will take Maraviroc 300 mg at 6-8AM. The capsules will be supplied by the hospital pharmacies and used under IRB approval as required.

The efficacy, safety, and biomarker evaluations that will be performed at each visit of the treatment phase and are described in the Time and Event Schedule.

Post-treatment phase

The efficacy and safety evaluations that will be performed at each visit during the post-treatment phase are described in the Time and Event Schedule.

All subjects who receive at least 1 dose of study medication should have 2 follow up visits conducted at 4 and 8 weeks after the last dose of study medication. The follow up visits at weeks 3, 5, 8, 9 will be telephone contacts. The follow up visit at 4 and 8 weeks after the last dose of study medication are clinic visits. Any clinically significant abnormalities persisting at the end of the study will be followed by the investigator until resolution or until a clinically stable endpoint is reached. All adverse events and special reporting situations, whether serious or non-serious, will be reported until completion of the subject's last study-related procedure (which may include contact for follow-up of safety).

TIME AND EVENTS SCHEDULE (Table S1)

| **Week 14 (4 weeks after last dose, Week 18 (8 weeks after last dose)** | **Telephone contact (week 3, 5, 8, 9)** | **week 4, week 7, week 10** | **Week 2** | **Day 1 (can be the same as screening)** | **Screening** | **Participant Assessment** |
| --- | --- | --- | --- | --- | --- | --- |
|  |  |  |  |  | **X** | Informed consent |
|  |  |  |  |  | **X** | Inclusion / Exclusion criteria |
|  |  |  |  |  | **X** | Medical history, demographics |
|  |  | **X** | **X** | **X** |  | Study Drug Administration |
|  |  |  |  |  |  | Safety Assessments |
| **X** |  | **X** | **X** | **X** | **X** | Physical examination |
|  |  | **X** | **X** | **X** | **X** | Body weight |
|  |  | **X** | **X** | **X** | **X** | Vital signs |
|  |  | **X** | **X** | **X** | **X** | 12-lead ECG |
|  |  |  |  |  | **X** | Carotid Doppler |
|  |  |  |  |  |  | Clinical Laboratory assessments |
|  |  | **X** | **X** |  | **X** | Hematology, Chemistry |
|  |  | **X** | **X** |  | **X** | Serum cytokines |
|  |  |  |  |  |  | Efficacy assessments |
| **X** |  | **X** | **X** | **X** | **X** | MADRS (7-day recall) |
| **X** |  |  |  | **X** |  | GDS |
|  |  | **X** | **X** | **X** | **X** | CGI-I + CGI-S |
| **X** |  | **X** | **X** | **X** | **X** | QIDS-SR16 |
|  |  | **X** | **X** | **X** | **X** | PGI-S |
| **X** |  | **X** | **X** | **X** | **X** | PGI-C |
| **X**  (for suspected Dementia) |  |  |  |  | **X** | GAD-7 (7-day recall) |
| **X** |  | **X (week 10)** |  |  | **X** | MoCA |
| **X** |  | **X (week 10)** |  | **X** |  | Computerized cognitive test battery (Neurotrax) |
| **X** |  |  |  | **X** |  | SIS |
| **X** |  | **X** |  | **X** |  | ADL |
| **X** |  |  |  | **X** |  | RNL |
|  |  |  |  |  |  | Ongoing Subject Review |
| **X** | **X** | **X** | **X** | **X** |  | Concomitant therapy^2^ |
| **X** | **X** | **X** | **X** | **X** |  | Adverse events^3^ |

^1^Visit window: Day 15 (+/- 1 day), Day 29, 43, 57 and 71 can occur +/- 3 days.

^2^Concomitant therapies must be recorded throughout the study beginning with signing of the informed consent until the last follow up visit.

^3^All adverse events and special reporting situations, whether serious or non-serious, will be reported from the time a signed and dated informed consent form is obtained until completion of the subject's last study-related procedure (which may include contact for follow-up of safety.

Abbreviations: **ADL** = the activities of daily living; **CGI-I =** clinical global impression – improvement; **CGI-S** = Clinical Global Impression – Severity (S); **ECG** = electrocardiogram; **GAD-7** = Generalized Anxiety Disorder 7-item scale; **GDS =** Geriatric depression scale; **MADRS** = Montgomery Asberg Depression Rating Scale; **MoCA** = Montreal Cognitive Assessment**; PGI-C** = Patient Global Impression of Change; **PGI-S** = Patient Global Impression – Severity; **RNL** = Reintegration to Normal Living Index; **SIS** = stroke impact scale (SIS).

**QIDS-SR16** = 16-item Quick Inventory of Depressive Symptoms- Self Report.

Laboratory work up: Blood samples for: cell blood count, lipids profile, liver and kidney function, inflammatory markers: highly sensitive c-reactive protein (hs-CRP), tumor necrosis factor (TNF)-alpha, interleukin-6 (IL-6), IL-10, IL-1 β, IL-2, IL-17A. Cytokine level will be determined using ELISA (R&D Systems, USA) and ProcartaPlex™ Multiplex Immunoassay (Affymtrix eBioscience) at the research laboratory for Stroke and dementia, Tel Aviv Sourasky Medical Center.

**Follow up assessment will be performed on week 2 and weeks 4, 7, 10, 14 and 18 and will include:**

Physical exam, vital signs, computerized cognitive tests (at week 10), questionnaires, record of AEs related or not related to the medication assignment, pill counts, blood samples.

**Interventions:**

Our participants will be free of hepatic, as well as renal impairment. The recommendation from the FDA is to obtain liver function tests prior to starting the drug and again should symptoms such as rash or hepatitis occur. This rather rare adverse reaction tends to occur at about one month after starting the medication (in less than 4% on the drug or placebo), so the investigators will obtain a bilirubin and transaminases at the planned 1-month follow up.

Adverse events related to the medication assignment, to rehabilitation practice or to other causes will be adjudicated by the safety committee with input from the principal investigator.

Patients will be contacted weekly by phone to help maintain interest in the trial, assure use of the assigned medication, and ask about possible adverse reactions. The coordinator will obtain this information and provide the feedback using a standard script and checklist.

**Quality control and analysis:**  All patient records and specimens will be tracked in a manner consistent with Good Clinical Practice by a quality-controlled, auditable, and appropriately validated laboratory information management system, to ensure compliance with data confidentiality as well as adherence to authorized use of specimens as specified in this protocol and in the Informed Consent Form.

**Statistical Analysis**

Safety analysis (Primary Endpoint): All safety data will be summarized in appropriate tables.

AEs will be coded according to coding dictionaries (MedDRA version 22.0 or higher) and presented in tables by System Organ Class (SOC) and Preferred Term (PT) and by treatment group. Safety will be also assessed by evaluating findings of physical examinations, vital signs, clinical laboratory test results, concomitant medications by treatment group. The changes from baseline in vital signs, clinical laboratory tests results will be displayed.

**Efficacy Assessments**

Primary outcomes analyses:

Stage 1- The Paired T-test or Signed rank test for two means (as is appropriate) will be applied for testing the statistical significance of the difference in depressive scores and in functional scores from baseline to week 10 and week 18.

Secondary outcome analyses:

Stage 1 - The Paired T-test or Signed rank test for two means (as is appropriate) will be applied for testing the statistical significance of the difference in scores from baseline to week 10 and week 18.

Pairwise comparisons of the study day assessments to baseline for significant main effects and interactions will be performed with Bonferroni corrections for multiple comparisons.

the effect of Maraviroc over time will be analyzed with repeated measures ANOVAs, with time as a within-subjects factor and depression scores (as measured by the MADRS) as the dependent variables. Associations between numeric variables will be determined using Spearman’s rank correlation analysis (coefficient estimate r).

Response rate will be defined as a ≥ 50% decrease from baseline depression scale scores to the trial endpoint. Significance will be evaluated at the α =.05 level, two-tailed, after Greenhouse-Geisser corrections.

**SAFETY PLAN**

Several measures will be taken to ensure the safety of patients participating in this study. Eligibility criteria have been designed to exclude patients at higher risk for toxicities. Patients will undergo safety monitoring during the study, including assessment of the nature, frequency, and severity of AEs. Investigators will assess the occurrence of AEs and serious AEs at all patient evaluation time-points during the study. All AEs and serious AEs, whether volunteered by the patient, discovered by study personnel during questioning, or detected through physical examination, laboratory test, or other means, will be recorded in the patient’s medical record and on the appropriate case report forms, as well as appropriately reported.

**References**

1. Broomfield NM, et al. Depression and anxiety symptoms post-stroke/TIA: Prevalence and associations in cross-sectional data from a regional stroke registry. BMC Neurol 14: 198, 2014
2. Robinson RG, Am J Psychiatry. 2016 Mar 1;173(3):221-31
3. Morris PL, Am J Psychiatry. 1993 Jan;150(1):124-9
4. Ayerbe L, Br J Psychiatry, 2013 Jan; 202(1):14-21
5. Tene O, J Clin Psychiatry. 2016 May;77(5):673-80
6. [FOCUS Trial Collaboration](https://www.ncbi.nlm.nih.gov/pubmed/?term=FOCUS%20Trial%20Collaboration%5BCorporate%20Author%5D). Effects of fluoxetine on functional outcomes after acute stroke (FOCUS): a pragmatic, double-blind, randomised, controlled trial. [Lancet.](https://www.ncbi.nlm.nih.gov/pubmed/30528472) 2019 Jan 19;393(10168):265-274.
7. [Kim JS](https://www.ncbi.nlm.nih.gov/pubmed/?term=Kim%20JS%5BAuthor%5D&cauthor=true&cauthor_uid=28012485), [Lee EJ](https://www.ncbi.nlm.nih.gov/pubmed/?term=Lee%20EJ%5BAuthor%5D&cauthor=true&cauthor_uid=28012485), [Chang DI](https://www.ncbi.nlm.nih.gov/pubmed/?term=Chang%20DI%5BAuthor%5D&cauthor=true&cauthor_uid=28012485), [Park JH](https://www.ncbi.nlm.nih.gov/pubmed/?term=Park%20JH%5BAuthor%5D&cauthor=true&cauthor_uid=28012485), [Ahn SH](https://www.ncbi.nlm.nih.gov/pubmed/?term=Ahn%20SH%5BAuthor%5D&cauthor=true&cauthor_uid=28012485), [Cha JK](https://www.ncbi.nlm.nih.gov/pubmed/?term=Cha%20JK%5BAuthor%5D&cauthor=true&cauthor_uid=28012485), [Heo JH](https://www.ncbi.nlm.nih.gov/pubmed/?term=Heo%20JH%5BAuthor%5D&cauthor=true&cauthor_uid=28012485), [Sohn SI](https://www.ncbi.nlm.nih.gov/pubmed/?term=Sohn%20SI%5BAuthor%5D&cauthor=true&cauthor_uid=28012485), [Lee BC](https://www.ncbi.nlm.nih.gov/pubmed/?term=Lee%20BC%5BAuthor%5D&cauthor=true&cauthor_uid=28012485), [Kim DE](https://www.ncbi.nlm.nih.gov/pubmed/?term=Kim%20DE%5BAuthor%5D&cauthor=true&cauthor_uid=28012485), [Kim HY](https://www.ncbi.nlm.nih.gov/pubmed/?term=Kim%20HY%5BAuthor%5D&cauthor=true&cauthor_uid=28012485), [Kim S](https://www.ncbi.nlm.nih.gov/pubmed/?term=Kim%20S%5BAuthor%5D&cauthor=true&cauthor_uid=28012485), [Kwon DY](https://www.ncbi.nlm.nih.gov/pubmed/?term=Kwon%20DY%5BAuthor%5D&cauthor=true&cauthor_uid=28012485), [Kim J](https://www.ncbi.nlm.nih.gov/pubmed/?term=Kim%20J%5BAuthor%5D&cauthor=true&cauthor_uid=28012485)14, [Seo WK](https://www.ncbi.nlm.nih.gov/pubmed/?term=Seo%20WK%5BAuthor%5D&cauthor=true&cauthor_uid=28012485), [Lee J](https://www.ncbi.nlm.nih.gov/pubmed/?term=Lee%20J%5BAuthor%5D&cauthor=true&cauthor_uid=28012485), [Park SW](https://www.ncbi.nlm.nih.gov/pubmed/?term=Park%20SW%5BAuthor%5D&cauthor=true&cauthor_uid=28012485), [Koh SH](https://www.ncbi.nlm.nih.gov/pubmed/?term=Koh%20SH%5BAuthor%5D&cauthor=true&cauthor_uid=28012485), [Kim JY](https://www.ncbi.nlm.nih.gov/pubmed/?term=Kim%20JY%5BAuthor%5D&cauthor=true&cauthor_uid=28012485), [Choi-Kwon S](https://www.ncbi.nlm.nih.gov/pubmed/?term=Choi-Kwon%20S%5BAuthor%5D&cauthor=true&cauthor_uid=28012485); [EMOTION investigators](https://www.ncbi.nlm.nih.gov/pubmed/?term=EMOTION%20investigators%5BCorporate%20Author%5D). Efficacy of early administration of escitalopram on depressive and emotional symptoms and neurological dysfunction after stroke: a multicentre, double-blind, randomised, placebo-controlled study. [Lancet Psychiatry.](https://www.ncbi.nlm.nih.gov/pubmed/28012485) 2017 Jan;4(1):33-41.
8. Preston TC, Shelton RC. [Treatment resistant depression: strategies for primary care.](https://www.ncbi.nlm.nih.gov/pubmed/23712721) Curr Psychiatry Rep. 2013 Jul;15(7):370
9. Trivedi MH. [Major depressive disorder: remission of associated symptoms.](https://www.ncbi.nlm.nih.gov/pubmed/16848674) J Clin Psychiatry. 2006;67 Suppl 6:27-32. Review
10. [Maes M](https://www.ncbi.nlm.nih.gov/pubmed/?term=Maes%20M%5BAuthor%5D&cauthor=true&cauthor_uid=19085093), et al.  inflammatory & neurodegenerative (I&ND) hypothesis of depression: leads for future research and new drug developments in depression. [Metab Brain Dis.](https://www.ncbi.nlm.nih.gov/pubmed/?term=The+inflammatory+%26+neurodegenerative+(I%26ND)+hypothesis+of+depression%3A+Leads+for+future+research+and+new+drug+developments+in+depression) 2009 Mar;24(1):27-53
11. Warden D et al, Current Psychiatry Reports 2007, 9:449-459
12. Mead, G. E. et al. Selective serotonin reuptake inhibitors (SSRIs) for stroke recovery. Cochrane Database Syst. Rev. 11, CD009286 (2012).
13. Duman RS, et al. Nat Med.2016 Mar;22(3):238-49
14. Raedler TJ, et al. (2011) Curr Opin Psychiatry 24, 519-525
15. Oglodek EA, et al. (2014) Pharmacol Rep
16. Raison CL, et al. Trends Immunol. 2006;27:24–31
17. Zunszain PA, et al. Prog Neuropsychopharmacol Biol Psychiatry. 2011 Apr 29;35(3):722-9).
18. Zhou M, Greenhill S, Huang S, Silva TK, Sano Y, Wu S, Cai Y, Nagaoka Y, Sehgal M, Cai DJ, Lee YS, Fox K, Silva AJ. CCR5 is a suppressor for cortical plasticity and hippocampal learning and memory. Elife. 2016 Dec 20;5.
19. [Mondal S](https://www.ncbi.nlm.nih.gov/pubmed/?term=Mondal%20S%5BAuthor%5D&cauthor=true&cauthor_uid=31043478), et al Low-Dose Maraviroc, an Antiretroviral Drug, Attenuates the Infiltration of T Cells into the Central Nervous System and Protects the Nigrostriatum in Hemiparkinsonian Monkeys. [J Immunol.](https://www.ncbi.nlm.nih.gov/pubmed/?term=maraviroc+parkinson) 2019 May 1.
20. Joy MT, Ben Assayag E, Shabashov-Stone D, Liraz-Zaltsman S, Mazzitelli J, Arenas M, Abduljawad N, Kliper E, Korczyn AD, Thareja NS, Kesner EL, Zhou M, Huang S, Silva TK, Katz N, Bornstein NM, Silva AJ, Shohami E, Carmichael ST. CCR5 Is a Therapeutic Target for Recovery after Stroke and Traumatic Brain Injury. Cell. 2019 Feb 21;176(5):1143-1157.e13.
21. Ogłodek EA, Szota AM, Just MJ, Mos´ DM, Araszkiewicz A. The MCP-1, CCL-5 and SDF-1 chemokines as pro-inflammatory markers in generalized anxiety disorder and personality disorders. Pharmacol Rep 2015;67(1):85–9
22. [Ogłodek EA](https://www.ncbi.nlm.nih.gov/pubmed/?term=Og%C5%82odek%20EA%5BAuthor%5D&cauthor=true&cauthor_uid=26481549), [Szota AM](https://www.ncbi.nlm.nih.gov/pubmed/?term=Szota%20AM%5BAuthor%5D&cauthor=true&cauthor_uid=26481549), [Moś DM](https://www.ncbi.nlm.nih.gov/pubmed/?term=Mo%C5%9B%20DM%5BAuthor%5D&cauthor=true&cauthor_uid=26481549), [Araszkiewicz A](https://www.ncbi.nlm.nih.gov/pubmed/?term=Araszkiewicz%20A%5BAuthor%5D&cauthor=true&cauthor_uid=26481549), [Szromek AR](https://www.ncbi.nlm.nih.gov/pubmed/?term=Szromek%20AR%5BAuthor%5D&cauthor=true&cauthor_uid=26481549). Serum concentrations of chemokines (CCL-5 and CXCL-12), chemokine receptors (CCR-5 and CXCR-4), and IL-6 in patients with posttraumatic stress disorder and avoidant personality disorder. 2015 Dec;67(6):1251-8.
23. Oglodek EA, Szota A, Just MJ, Mos D, Araszkiewicz A (2014) Comparison of chemokines (ccl-5 and sdf-1), chemokine receptors (ccr-5 and cxcr-4) and il-6 levels in patients with different severities of depression. Pharmacol Rep 66, 920-926
24. [Rasenick MM](https://www.ncbi.nlm.nih.gov/pubmed/?term=Rasenick%20MM%5BAuthor%5D&cauthor=true&cauthor_uid=27968725). Depression and Adenylyl Cyclase: Sorting Out the Signals. [Biol Psychiatry.](https://www.ncbi.nlm.nih.gov/pubmed/27968725) 2016 Dec 1;80(11):812-814.
25. Price T, Brust TF. [Adenylyl cyclase 7 and neuropsychiatric disorders: A new target for depression?](https://www.ncbi.nlm.nih.gov/pubmed/30904753) Pharmacol Res. 2019 May;143:106-112
26. [Yinan Wu](https://www.ncbi.nlm.nih.gov/pubmed/?term=Wu%20Y%5BAuthor%5D&cauthor=true&cauthor_uid=30618863)  et al. Mechanisms and Therapeutic Targets of Depression After Intracerebral Hemorrhage. [Front Psychiatry](https://www.ncbi.nlm.nih.gov/pmc/articles/PMC6304443/). 2018; 9: 682.
27. Tiraboschi JM, Niubo J, Curto J, Podzamczer D. Maraviroc concentrations in cerebrospinal fluid in HIV-infected patients. J Acquir Immune Defic Syndr 2010;55:606–609
28. Yilmaz A, Watson V, Else L, Gisslen M. Cerebrospinal fluid maraviroc concentrations in HIV-1 infected patients.AIDS. 2009;23:2537–2540
29. Abel S, Davis JD, Ridgway CE, Hamlin JC, Vourvahis M. Pharmacokinetics, safety and tolerability of a single oral dose of maraviroc in HIV-negative subjects with mild and moderate hepatic impairment. Antivir Ther. 2009;14(6):831–837.
30. Dorr P, Westby M, Dobbs S, Griffin P, Irvine B, Macartney M, Mori J, Rickett G, Smith-Burchnell C, Napier C, Webster R, Armour D, Price D, Stammen B, Wood A, Perros M. Maraviroc (UK-427,857), a potent, orally bioavailable, and selective small-molecule inhibitor of chemokine receptor CCR5 with broad-spectrum anti-human immunodeficiency virus type 1 activity. Antimicrob Agents Chemother (2005) 49:4721–32.
31. Abel S, van der Ryst E, Rosario MC, Ridgway CE, Medhurst CG, Taylor-Worth RJ, Muirhead GJ. Assessment of the pharmacokinetics, safety and tolerability of maraviroc, a novel CCR5 antagonist, in healthy volunteers. Br J Clin Pharmacol (2008) 65(S1):5–18
32. [Van Der Ryst](http://www.frontiersin.org/people/u/205769) E. Maraviroc - A CCR5 Antagonist for the Treatment of HIV-1 Infection. Front Immunol. 2015 Jun 5;6:277.
33. Lazzarin A, Revnes J, Molina JM, Valluri S, Mukwaya G, Heera J, Craig C, van der Ryst E, Sierra-Madero JG. The maraviroc expanded access program - safety and efficacy data from an open-label study. HIV Clin Trials. 2015 Jan-Feb;16(1):10-21.
34. Gulick RM, Lalezari J, Goodrich J, Clumeck N, DeJesus E, Horban A, Nadler J, Clotet B, Karlsson A, Wohlfeiler M, Montana JB, McHale M, Sullivan J, Ridgway C, Felstead S, Dunne MW, van der Ryst E, Mayer H, MOTIVATE Study Teams. Maraviroc for previously treated patients with R5 HIV-1 infection. N Engl J Med. 2008 Oct 2;359(14):1429-41.
35. Lazzarin A, Than S, Valluri SR, Heera J, Mukwaya G. Safety profile of maraviroc in patients coinfected with HIV-1 and hepatitis B or C included in the maraviroc expanded access program. HIV Clin Trials. 2012 Mar-Apr;13(2):83-9.
36. Kliper E, Ben Assayag E, Tarrasch R, Artzi M, Korczyn AD, Shenhar-Tsarfaty S, Aizenstein O, Hallevi H, Mike A, Shopin L, Bornstein NM, Ben Bashat D. Cognitive state following stroke: the predominant role of preexisting white matter lesions. PlosOne. 2014 Aug 25;9(8):e105461.
